# Analysis of Fruit and Vegetable Consumption by Children in School Canteens Depending on Selected Sociodemographic Factors

**DOI:** 10.3390/medicina55070397

**Published:** 2019-07-22

**Authors:** Edyta Łuszczki, Grzegorz Sobek, Anna Bartosiewicz, Joanna Baran, Aneta Weres, Katarzyna Dereń, Artur Mazur

**Affiliations:** 1 Institute of Nursing and Health Sciences, Faculty of Medicine, University of Rzeszow, 35-959 Rzeszow, Poland; 2 Institute of Physiotherapy, Faculty of Medicine, University of Rzeszow, 35-959 Rzeszow, Poland

**Keywords:** childhood nutrition, dietary patterns, food environment, food selection, fruit and vegetable

## Abstract

*Background and Objectives:* Eating habits acquired or changed during childhood are likely to track into adulthood. Due to the fact that nutritional behaviours are not so strongly formed among children, it is easier to change and develop them in children than in adults. The aim of this study was to assess the impact of selected sociodemographic factors affecting fruit and vegetable consumption (i.e., age, parents’ body mass index, parents’ level education, duration of breastfeeding, child’s time spent in front of computer/television) among children in school canteens. *Materials and Methods*: The sample consisted of 106 participants (52 girls, 54 boys) aged 6–12. The frequency of consuming fruits and vegetables at a school canteen was assessed using bar code cards for two weeks. Body composition estimates were obtained using a foot-to-foot bioelectrical impedance analysis, body height was measured using a stadiometer Seca 213. The questionnaire contained questions about selected factors which can have an influence on fruit and vegetable consumption. In the study group, 13.2% of participants were overweight and 17.9% were obese. *Results:* Our results showed a statistically significant relationship between age and fruit and vegetable consumption, and it increased with age in both sexes. *Conclusions:* Bearing in mind the various conditions discussed when shaping the eating habits of pre-school- and early-school-aged children, the importance of proper nutritional education should be stressed both among children and parents.

## 1. Introduction

Recent studies have indicated an increase in incidences of overweightness and obesity among children in Poland. Knai et al. [1] reported high levels of overweightness and obesity, with an increasing tendency among populations of adults and children, not only in Western Europe, but also in Eastern Europe. Another study [2] has shown that children’s and adolescents’ age-standardised mean BMIs increased globally and in most regions; although, in Eastern and Central Europe from 1975 to 2016, there was virtually no change in the BMIs of girls and boys. Overall, however, there was a sustained upward trend in Eastern and Central Europe, and other researchers have also noticed a continual growth trend in this region [3,4,5].

The risk of obesity is a global problem and children with obesity are more likely to also have obesity as adults [6,7,8,9]. A nutritious, balanced diet is an important factor in the prevention and treatment of excessive body weight. However, both children and adults rarely comply with dietary recommendations [10,11]. According to many research studies, individual risk factors with associations to overweightness and obesity include lack of physical activities, prolonged TV watching and playing computer games and frequent consumption of fast food and calorie-dense food items. Family-level risk factors include higher socioeconomic status and family history of obesity [3]. 

The level of vegetable and fruit intake is particularly low. In most American, Australian and European studies, children aged between two and eleven eat on average 2–3 servings of vegetables and fruits a day, despite the recommended 5 servings [10]. Recent European studies have shown that only 8.8% of children consume 5 servings of fruits and vegetables a day [11]. Numerous scientific experiments have shown that vegetables are the least liked food [12,13], and forcing children to try unpopular, unfamiliar dishes is not enough to increase the frequency of their consumption. Research confirms that the preferences and eating patterns of children are to a large extent a reflection of the food that has become known to them. Literature shows that the degree to which fruits and vegetables are present and easily available at home is positively correlated with the level of their consumption among school-aged children [14,15].

Several factors are known to influence children’s consumption of fruits and vegetables. Previous studies have reported that early feeding practices, including breastfeeding duration, may be related to fruit and vegetable intake in later life [16,17].

In the literature, there exist studies that indicate a correlation between vegetable consumption and mothers’ education level [18]. Research also shows that parents’ body mass index can also affect the child’s BMI and fruit and vegetable consumption [19,20]. The age of the child can also be important in the consumption of vegetables and fruit [21]. Television and computer viewing are hypothesized as contributing factors because of their documented role in encouraging consumption of highly advertised foods that may lead to the replacement of fruits and vegetables [22]. It is also a significant question whether giving children pocket money to buy additional food in a school shop can affect whether these children are less willing to later choose vegetables and fruits in the canteen during lunch. Children would not risk wasting money on new food choices or on fruits and vegetables for which there was no guarantee of a pleasant taste as opposed to a chocolate bar which would always taste the same [23,24].

School in Poland is a place where children, depending on their age, spend 4/5–8 hours daily ten months per year. During school hours they should drink and eat at least one meal. Eating at school should be considered in a broader context than only fulfilling physiological needs for nutrients and energy (i.e., time spent in front of a computer/television, giving child pocket money to buy food at school, parents’ level of education). In Poland, all students can benefit from school lunches in the school canteen, which are paid for on a monthly basis in advance, but participation in the school lunch program is not obligatory. The trend of only some of the students benefitting from school meals is continuing in Poland. The school canteen offers all students one type of meal including fruits and vegetables. Besides the canteen, children can buy food in the school shop. Most often, if the child does not eat lunch at school, he/she brings a sandwich/snacks from home, because school does not offer free snacks. Schools serve free meals for students coming from poor families. From 1 September 2015, the products offered in school shops changed, as a result of an amendment to the Act of 25 August 2006 on Food and Nutrition Safety. On 1 September 2015, new regulation concerning food provision in food shops at school and in vending machines as well as food-based standards for lunch were entered into force [25]. According to this act, schools cannot be a place for the sale of food products of unsatisfactory quality and which are not recommended in children’s diets. The assortment of foods and beverages sold in schools should comply with principles of sound nutrition. Some studies show that respondents reported lower shopping frequency after the changes were introduced [26]. After the regulation was passed, the number of children buying sweets and salty snacks dropped; however, the problem has not been completely eliminated. An analysis of another study demonstrated that the number of healthy products recommended for the age group increased only slightly [27].

A few reports on the impact of various factors on the consumption of fruits and vegetables during meals in school canteens are available in literature. However, they did not measure the consumption directly in school canteens. Therefore, the aim of the study was to assess the impact of selected factors affecting fruit and vegetable consumption by children aged 6–12 in school canteens.

## 2. Materials and Methods 

### 2.1. Subjects

The study included healthy children aged 6–12 with their parents during the school year 2015/2016. In order to select a school for the study, invitations were sent to all schools (1148 schools) in the Subcarpathian Province (southeast Poland). Those schools represent a similar profile of students and offered similar school lunch menus (public schools). From all those that consented, one school was selected for participation in the study (via randomized algorithm).

The inclusion criteria for participants in the study group were the daily consumption of lunches in the school canteen and no food intolerance/allergy; the exclusion criteria were missed lunches during the two weeks of the experiment and a food intolerance/allergy.

Invitations to participate in the study were directed to 168 parents or guardians of the examined children who were eating lunch in the school canteen. The consent of 122 people was obtained for participation in this study. Of those respondents, 16 were excluded from the study for the following reasons: intolerance for some products (*n* = 6), failure to return or complete the survey (*n* = 4) and refusal to participate in the study (*n* = 6). Ultimately, the study group consisted of 106 students (52 girls and 54 boys) aged 6–12. There were also 106 mothers and 105 fathers included in the study, and 105 children were from a two-parent home. The study was conducted after obtaining written consent from the participating children’s parents and the children themselves. All participants and parents were fully informed in writing and verbally about the nature of the study.

### 2.2. Assessments

#### 2.2.1. Anthropometric Measurements and Body Mass Index

Children’s anthropometric measurements were carried out in the Centre for Innovative Research in Medical and Natural Sciences (Rzeszow, Poland). All measurements were taken between 7:00 and 10:00 by experienced researchers.

Body height was measured to the nearest 0.5 cm using a portable stadiometer Seca 213. The measurements were performed under standard conditions, in an upright position, barefoot. Body weight was assessed with an accuracy of 0.1 kg using a body composition analyser (BC-420, Tanita). Body mass index (BMI) was calculated as weight (kg)/height (m^2^). Based on BMI values, the BMI percentile of the individual participants was calculated. Body mass index percentile charts specific for age, sex, and body height were used. Percentile charts which were developed within the framework of the Polish project entitled “Developing standards of blood pressure in children and adolescents in Poland, OLAF” were used [28]. Based on the BMI percentile values, underweight (<5th percentile), normal weight (between 5th and 85th percentile), overweight (BMI ≥ 85th percentile and < 95th percentile), or obesity (≥95th percentile) were determined. The definitions of underweight, normal weight, overweight, and obesity were based on the recommendations of the Centres for Disease Control and Prevention [29]. Parental weight and height measurements were based on self-report in the questionnaire. Body mass index classification for parents was carried out according to the WHO (World Health Organization) guidelines: underweight (<18.5 kg/m^2^), normal weight (between 18.5 and 24.99 kg/m^2^), overweight (between 25 and 29.99 kg/m^2^), and obesity (>30 kg/m^2^) [30].

#### 2.2.2. Bioelectrical Impedance

Body composition estimates were obtained using a Tanita device (BC-420) which uses foot-to-foot bioelectrical impedance analysis (BIA). All measurements were performed in the early morning according to the guidelines of the manufacturer with a frequency of 50 kHz. The bioelectrical impedance analysis method consists of measuring the impedance (electrical resistance, which consists of resistance and reactance) of tissue through which an electrical current of low intensity is passed (<1 mA) [31]. Measurements were taken after an overnight fast (for at least 8 h) because food or beverage consumption may decrease impedance by 4–15 Ω over a 2–4 h period after meals, representing an error smaller than 3% [32]. The percentage body fat (%FAT) was analysed.

#### 2.2.3. Questionnaires

The questionnaire used in the study covered basic information about the family background, family lifestyle, health and well-being and sociodemographic data. Parents or guardians of the examined children were requested to give written answers to the following: their date of birth, body height and body weight, number of people in the household, number of children, place of living, level of education (for both parents/guardians)—primary, secondary or higher education; diagnosed childhood diseases and intolerances/allergies, provision of pocket money to buy food at school (yes/no), duration of breastfeeding (as per the mother) for the examined child (in months) and time spent by child in front of a computer and television during the weekdays and weekends per day (less than 30 min, 30–60 min, 60–120 min, more than 120 min).

#### 2.2.4. The Course of the Study in the School Canteen

The frequency of eating fruits and vegetables at a school canteen was assessed using bar code cards. Each child had their own card with their unique ID number. The individual code of each child combined them with demographic data, such as age, class and sex. In the case of a child having a serving of vegetables and/or fruits (80 g or more) for lunch at the school canteen, the researcher scanned the code recording that amount and the data was stored on the computer disk. The evaluation of the frequency of eating fruits and vegetables in the school canteen lasted two weeks. 

Lunch in Polish school canteens contains portion of potatoes, rice or pasta and a piece of meat/fish/legumes given on one plate to children by a kitchen worker. Bowls with salad/fruits/vegetables were on the tables where the children consumed their meals. To eat the portion of salad/fruit/vegetables children had to put them on his/her plate. There was also a glass of water or tea offered to the children. There was only one type of meal for all children, without a choice. The menu was planned 10 days ahead, but children do not choose their meal from a buffet. Furthermore, there was one kind of salad/fruit/vegetable to choose from for all children, which was supplied and prepared before the lunch in the canteen kitchen. During the whole period covered by the study, children could put on their plates chosen salads, fruits, and vegetables. 

### 2.3. Statistical Analysis

Results of the study were developed using descriptive statistics: number—*n* (%), mean, median—Me, and standard deviation—SD. Both parametric and non-parametric tests were used to analyse the variables. The choice of the parametric test depended on fulfilling its basic assumptions, i.e., the conformity of the tested variable with normal distribution, which were verified by the Shapiro–Wilk test. The student’s *t*-test was used for normally distributed variables; alternatively, a non-parametric Mann–Whitney U test was used. The analysis of variables having the character of qualitative data was carried out with the Pearson chi-square test. The correlation of two variables was calculated with the Spearman rank correlation coefficient. Additionally, linear regression analysis was used to model a continuous variable–frequency of fruit and vegetable consumption. The factors were numeric or quantitative, which is why the analysis used the general regression model (GRM), which permits taking into account both numerical and qualitative variables as independent variables. Using the stepwise forward regression procedure, the selection of factors in a statistically significant way describing the level of vegetable and fruit consumption was made. The initial set of independent variables were the factors included in the analyses (age, sex, body fat percentage in children, children’s and parents’ body mass index, parents’ level of education, pocket money to buy food at school, duration of breastfeeding, time spent in front of computer/television by child on weekdays and weekends). Statistical significance was established as a *p*-value less than 0.05. Calculations were performed with Statistica 10.0 (StatSoft, Inc., Tulsa, OK, USA).

### 2.4. Ethics

The study was approved by the institutional Bioethics Committee at the University of Rzeszow from 2.6.2015 (Resolution No. 13/06/2015) and by all appropriate administrative bodies.

## 3. Results

### Characteristics of the Study Group

In the study group, 13.2% of participants were overweight and 17.9% with obesity. In the group of parents, 19.8% of mothers and 53.3% of fathers were overweight, and 6.6% and 14.3% with obesity, respectively. The descriptive characteristics of the study sample is shown below in Table 1.

The characteristics of fruit and vegetable consumption during the weeks covered by the study are presented in Table 2.

The influence of selected factors on the frequency of vegetable and fruit consumption in the school canteen are presented in Table 3 and Table 4.

In Table 3, the frequency of consumed fruit and vegetables and selected factors are presented. Children whose parents had secondary education consumed fruit and vegetables more often (*p* = 0.0430). 

Frequency of fruit and vegetable intake increased with age in both sexes (*p* = 0.0008). Analysis of the association between the results of the amount of consumed fruit and vegetables in school canteens and parents’ body mass showed a positive correlation between consumed fruits and vegetables and a mother’s BMI, indicating that higher BMI was associated with higher consumption of fruits and vegetables in school canteens (*p* = 0.0404) (Table 4).

A significant difference in screen time spent on school days was noted among participants; children who spent more time in front of a computer ate fruits and vegetables more often.

In the regression model, in which all independent variables were included, only the age of the child was statistically significant (Table 5 and Table 6). The mother’s BMI factor was not statistically significant, although the *p*-value was quite low (*p* < 0.10). In total, all these factors explained 15.5% of the variability in the level of fruit and vegetable consumption in the study group. Older children ate school meals containing fruits and vegetables 3.3% more often for each year of life, while the mother’s BMI value by 1 was the average increase in the level of vegetable and fruit consumption by 1.0%. 

## 4. Discussion

A few reports comparing the relationship between selected factors and fruit and vegetable consumption in school canteens among a population of healthy children are available in the literature. The presented issue is valuable as increasing children’s consumption of fruits and vegetables in the future seems to be the most important factor in weight reduction.

The epidemic of wealthy-civilization diseases, including obesity, is currently one of the greatest problems of modern medicine and societies with a high degree of socio-economic development. It is a medical, social and economic problem. The World Health Organization warns: there are over 110 million children in the world with excessive body mass [33]. There are many programmes in Poland being carried out in the area of nutrition and healthy eating education. These programmes offered to schools by various food producers, foundations, NGOs, as well as authoring programs developed in the schools themselves. The most popular nationwide program in Poland is the “milk glass programme”, which was also implemented at the school where the research was conducted. The programme “A Glass of Milk” was implemented in 2004 in schools in Poland. This program was developed in the frame of the Common Agricultural Policy established by the European Commission and aimed to increase the consumption of milk and dairy products [34]. According to our knowledge, this programme had no influence on fruit and vegetable consumption in the school canteen during the period of our study.

Continuous monitoring of obesity and overweightness is important for interventions aimed at preventing or reducing their complications [35]. To determine overweightness and obesity among children, weight–height indices and body weight patterns for a given sex and age were used. Some authors determined them by following such international tools as IOTF (International Obesity Task Force), WHO (World Health Organization), or American guidelines CDC (Centre Disease Control), the others opted for national criteria [36,37,38,39]. The body mass index (BMI) has also been used for many years. It is recommended by experts, among others the European Childhood Obesity Group (ECOG), and widely accepted and used in research in Poland and worldwide [40]. The BMI and body weight percentile charts with respect to age developed in the OLAF study have been used in our tests [28].

Overweightness and obesity were found in 31.1% of children in our study. Kowal et al. [41] examined children aged 3–18 from the city of Krakow in 1983, 2000 and 2010 and observed that the increase in the incidence of overweightness and obesity concerned mainly boys, while in girls it remained at a similar level. Meigen et al. [42] observed a significant increase in boys’ obesity compared to girls in 1999 and 2006 in Germany. Oblacińska et al. [43] found overweightness in 8.1–8.5% and obesity in 2.9–3.6% of boys aged 13–15. Among girls, they were 8.1–10.1% and 5.2–6.2%, respectively. In 2008/2009, Bac et al. [44] conducted an analysis of the nutritional status among 1499 students aged 6–13. They pointed out that the percentage of children with obesity increased in boys from 1.04% in 1971 to 7% in 2009, while in girls it increased from 0.20% to 3.6%, respectively.

Fruits and vegetables are the basis for healthy nutrition. They provide vitamins, minerals, fibre, as well as many important substances, such as plant sterols, flavonoids and antioxidants. Their daily intake helps to prevent non-infectious diseases such as cardiovascular disease, diabetes and cancer. The World Health Organization recommends the intake of more than 400 g of fruits and vegetables per day to improve overall health and reduce the risk of disease [45]. Low intake of vegetables is especially a persistent problem despite the increased awareness of the influence of their consumption on health [46,47,48]. It is estimated that 5.2 million deaths in the world in 2013 were caused by too low consumption of these products [49]. Unfortunately, research shows that in 21 European Union countries in 2009/2010, only 1 in 3 girls and 1 in 4 boys aged 15 years consumed at least one portion of fruit and vegetables per day [50]. Low fruit and vegetable intakes are a precursor of overweightness/obesity [51], while a high fruit/vegetable intake has beneficial effects on preventing excessive weight gain in adulthood [52,53]. There have been few studies which have examined dietary intake across many countries and BMI in children. A previous study of 34 (primarily European) countries participating in the 2001/2002 Health Behaviour in School-Aged Children Study reported that overweight status was not associated with the intake of fruits or vegetables [54]. Another prospective cohort study of 14,900 children demonstrated that neither fruit nor fruit juice intake predicted changes in BMI, but among boys, vegetable intake was inversely related to changes in BMI z-score [55]. In our study, we saw no significant correlations between children’s weight status and the consumption of fruits and vegetables in the school canteen.

The frequency of eating fruits and vegetables by children is influenced by many factors, including their availability at home and parents’ nutritional behaviour. In a meta-analysis of 18 studies on factors affecting fruit and vegetable consumption among people aged 6–18, such factors were distinguished as: sex, age, socio-economic status, fruit and vegetable consumption by parents and availability at home [56]. We found a statistically significant relationship between age and frequency of fruit and vegetable consumption, and it increased with age in both sexes. Zeinstra et al. [57] found a relationship between children’s cognitive development and their food preferences and perceptions; the rates of cognitive development relating to fruit and vegetable intake increased with age. Age-related differences in fruit and vegetable preferences have been found in other studies [58,59]. In our study, it was possible that when the children matured, their level of healthy behaviour increased. For this reason, children were able to consume more often fruits and vegetables during the meal. Furthermore, with increasing age, children’s caloric requirements also increase.

Parents’ education may also be an influential factor. A relationship between maternal education status and fruit and vegetable consumption has been shown before [60]; however, some studies have shown an association with fruit only [61,62] and others with vegetables only [63]. In a study conducted in 38 schools in Poland among 1255 students aged 9, factors influencing the consumption of fruits and vegetables by children were determined. The results indicated that the total daily intake was significantly higher in boys than in girls. The lowest was observed among children of parents with primary education. However, the highest consumption was in children whose parents had higher education [64]. Numerous scientific experiments confirm that parents’ education is a factor influencing fruit and vegetable consumption [65,66]. In our study, an increased frequency of vegetable and fruit intake during school meals was observed in children whose parents had secondary education. This may be due to the fact that parents with secondary education in Poland often have garden plots and children are familiar with the taste of vegetables and fruits from an early age. In the south-eastern part of Poland, people with lower education also often work in arable fields, which means that the availability of vegetables and fruits is greater in the home environment. It was reported that children’s perceived accessibility to fruits and vegetables at home is strongly correlated to parents’ fruit and vegetable intake [67]. Differences in study methods may partly explain these inconsistencies.

The correlation between the sex of the examined person and the amount of fruits and vegetables consumed was analysed by Rasmussen et al. [65] based on 49 scientific reports. In 27 of them it was pointed out that girls eat fruits and vegetables more often than boys. In 18, no differences were observed, while in the rest it was greater in boys. Dzielska et al. [68], in an earlier Polish study, reported that girls consumed fruits and vegetables more often than boys, but in our study, there was no statistically significant difference in the frequency of eating fruits and vegetables between girls and boys. Data in the literature also indicate a correlation between the consumption of mothers and pre-school children regarding the majority of food products, which indicates that one can improve the menu of younger people by means of interventions addressed to parents [69]. There is also a relationship between mothers’ health motivations and the feeding of children [70]. On this basis, it can be stated that parents’ attitudes and behaviours play a very important role in the process of children’s social learning.

In our study, children of overweight or obese mothers consumed fruit and vegetables more frequently than children of healthy weight mothers; this was found in the simple analysis, but this association was not statistically significant in the adjusted linear regression model. Consumption of fruits and vegetables by children is significantly correlated with the level of their consumption by parents [71,72]. Charlton et al. [73] reported that overweight and obese women were more likely than their normal weight counterparts to consume higher intakes of fruit and vegetables. However, observational data evaluating the relationship between fruit and vegetable intake and BMI provide inconclusive results [14,74,75]. The association between longer breastfeeding duration and higher fruit and vegetable intake later in life was found in previous studies [17,76]. Longer breastfeeding tends to be related to higher maternal educational level and higher diet quality, which could confound the association between breastfeeding and later fruit and vegetable intake in children. The association with breastfeeding was not explained by maternal fruit and vegetable intake. An explanation could be that familiarization of breastfed infants to flavours transmitted via breast milk from the range of flavours consumed by the mother leads to an increase in the acceptance of the flavours of fruits and vegetables. Results of a study by Mennella et al. [77] demonstrated that infants who had exposure to the flavour of carrots in either amniotic fluid or breast milk behaved differently in response to that flavour in a food base compared to control counterparts. In our study, there were no significant correlations between the duration of breastfeeding and the consumption of fruits and vegetables in the school canteen.

Finally, there appears to be a consensus among researchers about the potential influence of television and computer viewing on the eating patterns of children, including fruit and vegetable consumption, as well as on the need to take this into consideration in any nutrition education effort [78,79]. Research indicates that watching television is inversely correlated with the consumption of fruits and vegetables by children and may be caused by exposure to a large number of advertisements broadcast on television [80]. In our study, there was no statistically significant correlation between the frequency of fruit and vegetable intake and the time spent in front of TV, while a significant relationship was found between the time spent in front of the computer on school days and the consumption of fruits and vegetables. Children sitting in front of a computer monitor more on weekdays ate fruits and vegetables more often during lunch in the school canteen. One of the reasons may be lower exposure to advertising as in the case of television. Furthermore, studies show that computer games available to young children focus on using fruit and vegetable icons, may increase their visual exposure to a different variety of fruits and vegetables and may lead to positive impacts on acceptance [81,82,83]. In the future, it is necessary to specify the content that children watch on the computer (playing games, surfing the Internet, watching programs, etc.) and which ones most affect the consumption of vegetables and fruits.

The present study was able to document the impact of selected factors affecting the frequency of fruit and vegetable consumption in the participants studied. There are also a number of potential limitations of the study that need to be taken into account when interpreting the results. The primary study limitation was the relatively small sample size. Small sample can undermine the internal and external validity of a study and associations which do exist cannot be detected. Furthermore, selection bias might have led to reduced generalizability because only one school was included in the study. This study was also limited in geographic scope. Another study limitation could be that although children may have been offered fruits and vegetables, but they may not have consumed what was offered. Although screen time was shown in the questionnaires, residual confounding changes in screen time cannot be excluded. Residual confounding may be important because analyses have not accounted for major covariates such as physical activity and sedentary behaviour. Additionally, our study relied on parent-reported height and weight to calculate participants’ BMI, and retrospective data such as the question about the duration of breastfeeding years later could be recall bias. Response bias, such as social desirability, is common in self-reported questionnaires, and might have led to underestimation or overestimation of the present associations. Another study limitation was that parental control over eating behaviour was not assessed. Parental behaviour may impact the likelihood that children will exhibit addictive-like eating and the amount of food they consume. Also, it will be important for future research to examine the association between school canteen meals and student food purchases. The associations observed with school lunch fruit/vegetable consumption may be due to the residual confounding from exposure to other types of products. The interaction between price and other changes to the promotion, placement and availability of foods should also be examined. Lastly, this study had a cross-sectional study design, therefore, cause and effect cannot be determined. A possible strategy for future studies to minimize these limitations is to add more potential factors that may have an influence on fruit and vegetable consumption. Larger participant groups are needed to confirm the results and it should be replicated among a larger sample across more schools and regions. Research shows that the diet and eating habits of parents are the basic pattern on which their children’s diet is based. Parents choose foods from the family diet and they serve as food models that children learn to imitate. The intake of individual food products by children depends not only on the type of food at home, but also on the amount of food available, for example, at the school canteen. Parents’ influence is significant: it is reflected both by what is on the plate at home and what is chosen at school. As people responsible for the availability and diversity of food at home, they also influence what their children consume. In our study, we focused on several parental factors including parents’ BMI and parents’ education level; however, future research should focus more on the home environment and parents; diet.

## 5. Conclusions

Despite the limitations, our study allowed us to collect data on the frequency of fruit and vegetable consumption in school canteens during lunch, as well as to determine the impact of factors on their consumption in primary school students. It must be taken into account that fruit and vegetable consumption are clearly different behaviours, with different influencing factors. Our study shows that one of those factors is especially age. We found age-related differences in fruit and vegetable consumption and it increased with age in both sexes. Bearing in mind the various conditions discussed shaping the eating habits of pre-school- and early-school-age children, the importance of proper nutritional education should be stressed both among children and parents from the earliest years.

## Figures and Tables

**Table 1:** Characteristics of the study group.

Variable	Values
**Children**	
Age (years) ^a^	
6	42 (39.60)
7	19 (17.90)
8	5 (4.70)
9	6 (5.70)
10	11 (10.40)
11	12 (11.30)
12	11 (10.40)
Sex ^a^	
Boy	54 (50.90)
Girl	52 (49.10)
Body height ^b^	134.288 (15.16)
Body mass ^b^	33.5 (14.25)
Body mass category ^a^	
Underweight	6 (5.70)
Normal weight	67 (63.20)
Overweight	14 (13.20)
Obesity	19 (17.90)
Body composition ^b^	
%FAT	22.7 (5.60)
**Parents**	
Mother	Father
Age (years) ^b^	
38.1 (5.6)	40.9 (6.0)
BMI ^b^	
23.3 (3.5)	26.8 (3.3)
Body mass category ^a^	
Underweight	
5 (4.7)	2 (1.9)
Normal weight	
73 (68.9)	32 (30.5)
Overweight	
21 (19.8)	56 (53.3)
Obesity	
7 (6.6)	15 (14.3)
Level of education ^a^	
Primary education	
4 (3.8)	12 (11.4)
Secondary education	
66 (62.3)	70 (66.7)
Higher education	
36 (34)	23 (21.9)

BMI—body mass index. Data are expressed as: ^a^ *n* (%); ^b^ $\bar{x}$ (SD).

**Table 2:** Proportion of school canteen meals consumed during the study period which included vegetables and fruits.

Sex	Vegetable and Fruit Consumption							
	*n*	$\bar{x}$	Me	SD	*C*_25_	*C*_75_	Min	Max
Female	52	56%	60%	23%	40%	75%	0%	100%
Male	54	59%	60%	21%	40%	80%	20%	100%
Total	106	57%	60%	22%	40%	75%	0%	100%

$\bar{x}$—arithmetic mean; Me—median; SD—standard deviation; *C*_25_—the 25th percentile; *C*_75_—the 75th percentile.

**Table 3:** The influence of selected factors on the frequency of fruit and vegetable portions * consumed by children in the school canteens.

**Sex**	$\bar{x}$	**Me**	**SD**	***p*-Value**
Female	56%	60%	23%	0.4985
Male	59%	60%	21%	
**BMI classification**	$\bar{x}$	**Me**	**SD**	***p*-Value**
Underweight	64%	63%	23%	0.8899
Normal weight	57%	60%	20%	
Overweight	60%	60%	26%	
Obesity	55%	40%	27%	
**Duration of breastfeeding**	$\bar{x}$	**Me**	**SD**	***p*-Value**
<6 months	58%	60%	23%	0.8158
>6 months	57%	60%	22%	
**Parent education level**	$\bar{x}$	**Me**	**SD**	***p*-Value**
Secondary education	60%	60%	20%	0.0430 **
Higher education	52%	50%	25%	
**Pocket money to buy food at school**	$\bar{x}$	**Me**	**SD**	***p*-Value**
yes	58%	50%	24%	0.9448
no	57%	60%	22%	

* One portion was defined as 80 g fruit/vegetable; ** indicates significant values (*p* < 0.05). $\bar{x}$—arithmetic mean; Me—median; SD—standard deviation.

**Table 4:** Spearman correlation coefficients between selected factors and the consumption of vegetables and fruits for the entire population of children and divided by gender.

Factors	Sex of Child		
	Female	Male	Total
	Frequency of Fruit and Vegetable Consumption		
Age	0.30 (*p* = 0.0316 *)	0.34 (p = 0.0125 *)	0.32 (*p* = 0.0008 ***)
Z-score BMI	−0.22 (*p* = 0.1229)	0.01 (*p* = 0.9526)	−0.08 (*p* = 0.3877)
%FAT	−0.26 (*p* = 0.0636)	−0.11 (*p* = 0.4222)	−0.16 (*p* = 0.0913)
Mother’s BMI	0.10 (*p* = 0.4601)	0.29 (*p* = 0.0311 *)	0.20 (*p* = 0.0404 *)
Father’s BMI	0.22 (*p* = 0.1139)	−0.05 (*p* = 0.7013)	0.09 (*p* = 0.3425)
Screen time at the computer, weekdays (minutes/day)	0.10 (*p* = 0.4589)	0.28 (*p* = 0.0405 *)	0.18 (*p* = 0.0434 *)
Screen time at the computer, weekend (minutes/day)	0.04 (*p* = 0.7618)	0.17 (*p* = 0.2252)	0.11 (*p* = 0.2442)
TV on weekdays (minutes/day)	0.07 (*p* = 0.6351)	0.08 (*p* = 0.5868)	0.07 (*p* = 0.5066)
TV at the weekend (minutes/day)	0.00 (*p* = 0.9813)	0.05 (*p* = 0.7308)	0.04 (*p* = 0.6572)

* Indicates significant values (*p* < 0.05); *** *p* ≤ 0.001.

**Table 5:** Results of the general regression model for frequency of fruit and vegetable consumption (independent variables were selected by forward stepwise regression procedure).

Independent Variables	Frequency of Fruit and Vegetable Consumption		
	*R*^2^ = 15.5%; *F* = 9.5; *p* = 0.0002 ***		
	*B*	*p*	*β*
Age	3.3%	0.0003 ***	0.339
Mother’s BMI	1.0%	0.0716	0.166

*R*^2^—coefficient of determination; *F*—test statistic and significance of regression model (*p*); *B*—regression coefficient; *p*—significance of regression coefficient; *β*—standardize regression coefficient; *** *p* ≤ 0.001.

**Table 6:** Statistical significance of factors in the regression model with all explanatory variables.

Explanatory Variables	*p*
Age	0.0001 ***
Z-score BMI	0.7820
%FAT	0.2472
Sex	0.9024
Pocket money to buy food at school	0.2225
Duration of breastfeeding	0.1481
TV on weekdays	0.9927
TV at the weekend	0.9216
Screen time at the computer (weekdays)	0.5356
Screen time at the computer (weekend)	0.3195
Mother’s BMI	0.1480
Father’s BMI	0.9708

*p*—significance of the regression coefficient; *** *p* ≤ 0.001.

## Abbreviations

BIA	bioelectrical impedance analysis
BMI	body mass index
CDC	Centre Disease Control
ECOG	European Childhood Obesity Group
IOTF	International Obesity Task Force
Me	median
SD	standard deviation
WHO	World Health Organization
